# Clinical Manifestations and Mortality Predictors of COVID-19 in Patients Undergoing Chronic Hemodialysis: A Retrospective Cohort Study from Romania

**DOI:** 10.3390/jcm15031067

**Published:** 2026-01-29

**Authors:** Oana Nicolescu, Mihaela Magdalena Mitache, Andrei Mitache, Adelina-Gabriela Niculescu, Dragos Garofil, Victor Dan Eugen Strambu, Daniel Cochior, Elena Rusu, Cosmin Moldovan, Sorin Tudorache, Ioana Ruxandra Poiana, Dan Spinu, Alice Elena Munteanu, Marian Necula, Corneliu Ovidiu Vrancianu, Ana Maria Alexandra Stănescu

**Affiliations:** 1 Faculty of Medicine, “Carol Davila” University of Medicine and Pharmacy, 020021 Bucharest, Romania; droananicolescu@yahoo.com (O.N.); dragos.garofil@umfcd.ro (D.G.); victor.strambu@umfcd.ro (V.D.E.S.); ioana-ruxandra.poiana@umfcd.ro (I.R.P.); arsenie.spinu@umfcd.ro (D.S.); alexandra.stanescu@umfcd.ro (A.M.A.S.); 2 Faculty of Medicine, University Titu Maiorescu Bucharest, Gheorghe Petrașcu 67A, 031592 Bucharest, Romania; mitache.ad@gmail.com (A.M.); cochiordaniel@gmail.com (D.C.); elenarusu98@yahoo.com (E.R.); moldovan.cosmin@gmail.com (C.M.); soryntudorache@yahoo.com (S.T.); dralicemunteanu@gmail.com (A.E.M.); 3 Public Health Directorate, Avrig 72-74, District 2, 021578 Bucharest, Romania; 4 Research Institute of the University of Bucharest—ICUB, University of Bucharest, Șoseaua Panduri 90, District 5, 050663 Bucharest, Romania; adelina.niculescu@upb.ro (A.-G.N.); ovidiu.vrancianu@incdsb.ro (C.O.V.); 5 Department of Science and Engineering of Oxide Materials and Nanomaterials, National University of Science and Technology POLITEHNICA Bucharest, Splaiul Independenței 313, District 6, 060042 Bucharest, Romania; 6 Dr. Carol Davila Clinical Hospital of Nephrology, Calea Griviței 4, 010731 Bucharest, Romania; 7 Department of Urology, Central Military Hospital, Calea Plevnei 134, 010825 Bucharest, Romania; 8 Army Cardiovascular Disease Center, Central Military Emergency University, Calea Plevnei 134, 010825 Bucharest, Romania; 9 National Institute of Research and Development for Biological Sciences, 296 Splaiul Independentei, District 6, 060031 Bucharest, Romania; marian.necula@incdsb.ro; 10 Faculty of Administration and Business, University of Bucharest, 030018 Bucharest, Romania; 11 Doctoral School, “Carol Davila” University of Medicine and Pharmacy, Eroii Sanitari 8, District 5, 050474 Bucharest, Romania; 12 Academy of Romanian Scientists AOSR, Ilfov Street 3, District 5, 030167 Bucharest, Romania

**Keywords:** COVID-19, hemodialysis patients, retrospective study, clinical manifestations, prognostic factors

## Abstract

**Background/Objectives**: Patients undergoing chronic hemodialysis are at increased risk of severe COVID-19 outcomes. This study aimed to evaluate the clinical characteristics and prognostic factors associated with mortality in hemodialysis patients infected with SARS-CoV-2. **Methods**: We conducted a retrospective study including 130 chronic hemodialysis patients diagnosed with COVID-19 and admitted to a nephrology unit between March 2020 and April 2021. Demographic data, comorbidities, clinical manifestations, hospitalization duration, and outcomes were analyzed using univariate and multivariate statistical methods. **Results**: The cohort was predominantly male (64.6%), with a mean age of 64.0 ± 13.9 years. The mean hospitalization duration was 13.6 ± 9.7 days. Cardiovascular disease, chronic respiratory disease, dyspnea at presentation, and hospital-origin admission were significantly associated with mortality. While diabetes mellitus and hypertension were highly prevalent, they did not independently predict mortality after adjustment. Overall mortality was 34.6%, particularly among older patients with multiple comorbidities. **Conclusions**: COVID-19 infection is associated with substantial morbidity and mortality among patients on chronic hemodialysis. Early identification of high-risk patients based on clinical presentation and comorbidity profile may support timely intervention and improved outcomes in this vulnerable population.

## 1. Introduction

The coronavirus disease 19 (COVID-19) pandemic has been one of the most acute global crises in modern history, being marked by a significant burden on healthcare systems worldwide and leading to substantial morbidity and mortality. Caused by the severe acute respiratory syndrome coronavirus 2 (SARS-CoV-2), COVID-19 is now recognized as a complex multisystem disorder with intricate, interrelated pathophysiological mechanisms extending far beyond the respiratory tract [1,2,3].

Initial symptoms of COVID-19 closely resemble those of influenza, typically exhibiting fever, cough, malaise, and dyspnea [4]. Since its emergence in late 2019, this viral disease has been mainly viewed as a respiratory illness due to its pronounced pulmonary manifestations and airborne transmission dynamics [3]. However, while most people exhibit mild, flu-like symptoms, certain categories of individuals develop life-threatening forms that can lead to multiorgan failure and death [5].

Disease severity depends on various factors, including viral load, systemic inflammation, the balance between innate and adaptive immune responses, and pre-existing comorbidities [5]. After the virus enters the host cells, innate immunity is activated to limit replication; however, excessive immune stimulation can trigger a hyperinflammatory state characterized by a “cytokine storm,” which precipitates widespread tissue damage and organ dysfunction [6,7]. Once inside the cells, the viral genome is replicated, and newly assembled virions are released into adjacent cells, perpetuating viral spread. The viral receptor, angiotensin-converting enzyme II (ACE2), is highly expressed in multiple organ systems—including the respiratory tract, heart, gastrointestinal tract, and kidneys—thereby enabling SARS-CoV-2 to exert a systemic impact [1].

Among extrapulmonary organs, the kidneys are particularly susceptible to SARS-CoV-2 due to their high expression of the ACE2 receptor, which facilitates direct viral entry into renal epithelial cells [8]. Especially, patients with end-stage renal disease (ESRD) present an unfavorable combination of factors (e.g., chronic inflammation, uremic toxins, and immune dysfunction) that exacerbate infection susceptibility and increase the risk of severe outcomes [9]. Additional renal injuries can result from COVID-19 infection through direct (e.g., infection of renal tubular cells and induction of fibrotic remodeling) and indirect mechanisms (e.g., systemic inflammation, immune-cell infiltration, cytokine overproduction, angiotensin II pathway activation, complement dysregulation, endothelial dysfunction, and microangiopathy), as revealed by the findings of experimental studies performed on human-derived kidney. Moreover, the virus affects renal physiology through altered expression of ACE2, transmembrane serine protease 2 (TMPRSS2), and tissue proteinase L within kidney tissue, reinforcing both local and systemic damage [2,10,11,12,13].

Patients with chronic kidney disease (CKD), particularly those undergoing chronic hemodialysis, represent an exposed group to SARS-CoV-2 infection, exhibiting some of the highest reported mortality rates worldwide [2,14,15,16]. Multiple overlapping risk factors contribute to this increased vulnerability, including immunosuppression, advanced age, and frequent presence of associated comorbidities. Conditions like obesity, diabetes mellitus, hypertension, and cardiovascular disease are both prevalent and often more aggressive in the dialysis population compared to the general public [5,8,16,17,18].

Beyond intrinsic physiological factors, the logistics of dialysis treatment itself impose additional risks. Hemodialysis requires frequent, prolonged, face-to-face interactions with healthcare personnel and other patients, making social distancing practically impossible and increasing the likelihood of nosocomial transmission [16,18,19].

Moreover, numerous international studies have reported a severe course of COVID-19 in the dialysis population, with an increased rate of respiratory and cardiovascular complications, along with atypical manifestations involving the gastrointestinal tract and nervous system [20]. More severe COVID-19 forms can also be linked to the limited treatment options available for patients with renal dysfunction [4]. The unfavorable manifestations of the disease are reflected in large-scale registry data: according to the European Renal Association COVID-19 Database (ERACODA), the 28-day mortality among dialysis patients during the first pandemic wave reached 25%, with age, frailty, and comorbidity burden emerging as independent predictors of death [15].

Overall, the combination of impaired immunity, chronic systemic inflammation, metabolic alterations, and unavoidable exposure to healthcare settings places dialysis patients at uniquely high risk for severe COVID-19 outcomes. Understanding the clinical profile and prognostic determinants in this population is therefore essential for guiding early intervention, optimizing infection control in dialysis units, and reducing mortality through targeted management strategies.

While numerous studies have characterized COVID-19 outcomes in the general population, data focusing on critically ill patients undergoing chronic hemodialysis remain limited, particularly regarding prognostic indicators and outcome predictors in Eastern European cohorts. Therefore, the aim of the present study was to evaluate the clinical characteristics and identify factors associated with mortality in COVID-19 patients on chronic hemodialysis admitted to a specialized nephrology unit in Romania.

The present study adds relevant regional insight by focusing on an Eastern European cohort, where healthcare structures, patient demographics, and clinical pathways may differ from those reported in Western populations. Importantly, beyond descriptive confirmation of known risk factors, our analysis contributes to prognostic refinement by demonstrating that cumulative cardiorespiratory burden represents a more decisive determinant of mortality than isolated metabolic conditions, such as diabetes mellitus, in this specific population.

## 2. Materials and Methods

### 2.1. Study Design and Population

This retrospective study was conducted between March 2020 and April 2021 and included 130 dialysis patients undergoing chronic hemodialysis who were diagnosed with SARS-CoV-2 infection.

### 2.2. Dialysis Setting

**Figure 1:**
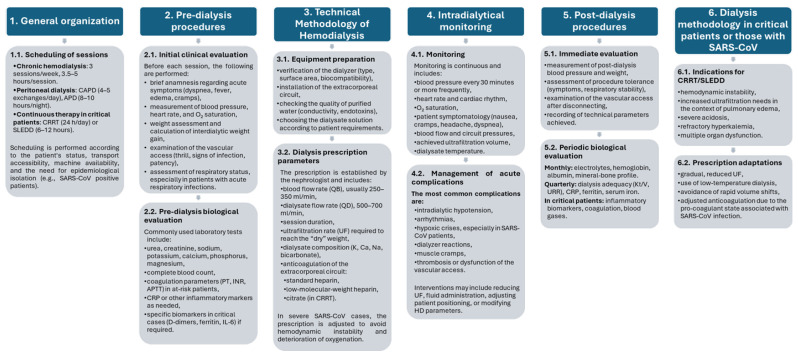
At-a-glance overview of the dialysis program structure.

The dialysis program followed a standardized structure (Figure 1), including pre-dialysis assessment, intradialytic monitoring, and post-dialysis procedures. Individual adaptations were performed based on patient comorbidities, volemic status, vascular access, and clinical severity of acute infection.

### 2.3. Data Collection

The investigation from the present study aimed to evaluate the clinical and demographic characteristics of this vulnerable group and to identify the risk factors associated with severe disease progression. Data collection was carried out through the analysis of medical records, systematically recording:

- Demographic characteristics: sex, age, residence (urban/rural), origin (hospital vs. dialysis center). These parameters allowed identification of patient distribution and its correlation with COVID-19 severity, as international studies have shown higher susceptibility to severe forms among elderly and male patients [21,22].
- Major comorbidities: cardiovascular diseases (hypertension, heart failure), metabolic disorders (diabetes mellitus, dyslipidemia), chronic respiratory diseases (COPD, asthma). International literature indicates that multiple comorbidities significantly increase the risk of prolonged hospitalization and mortality among dialysis patients infected with SARS-CoV-2 [23,24].
- Main clinical symptoms: fever, dyspnea, fatigue, cough, muscle pain, gastrointestinal symptoms. Clinical analyses have shown that dialysis patients may present atypical or subtle symptoms, which can delay diagnosis and prompt treatment [25].
- Length of hospitalization: the interval between admission and discharge or death, which helps assess healthcare burden and infection severity. International studies have reported more extended hospital stays among patients with chronic kidney disease and multiple comorbidities [25].
- Outcome: discharge with full recovery or death, an essential indicator for prognosis analysis and correlation with clinical and demographic risk factors.

### 2.4. Outcome Definition

The primary outcome was in-hospital mortality. Patients were classified as survivors (discharged alive) or non-survivors (death during hospitalization). Length of hospital stay was analyzed as a secondary outcome.

### 2.5. Statistical Analysis

Continuous variables were expressed as median (interquartile range) or mean ± standard deviation, as appropriate. Categorical variables were presented as counts and percentages. Comparisons between survivors and non-survivors were performed using the Wilcoxon rank-sum test for continuous variables and the chi-square or Fisher’s exact test for categorical variables. Logistic regression analysis was used to identify factors associated with in-hospital mortality. Odds ratios (ORs) with 95% confidence intervals (CIs) were calculated. Variables with clinical relevance and/or statistical significance in the univariate analysis were included in regression models. A *p*-value < 0.05 was considered statistically significant. Statistical analyses were performed using R software (version 4.5.2; R Foundation for Statistical Computing, Vienna, Austria).

Multivariable logistic regression analyses were performed using complete-case data; therefore, patients with missing values for any included covariates were excluded. As a result, the final multivariable model included 128 patients. Given the limited sample size relative to the number of covariates included, the multivariable logistic regression analysis was considered exploratory, and adjusted estimates were interpreted with caution due to the potential risk of overfitting. To assess multicollinearity among predictors, variance inflation factors (VIFs) were calculated for all variables included in the multivariable model.

In addition, dialysis vintage (defined as the duration of maintenance hemodialysis, in years) was calculated for patients with available data and categorized into predefined intervals (<1 year, 1–3 years, 3–5 years, 5–10 years, and >10 years). Patients with incomplete dialysis initiation records were excluded from this specific analysis. The association between dialysis vintage categories and in-hospital mortality was explored using descriptive analysis.

## 3. Results

### 3.1. Study Population and Baseline Characteristics

A total of 130 hemodialysis patients with confirmed COVID-19 were included. Of these, 83 patients survived and 45 died during hospitalization. Baseline demographic and clinical characteristics stratified by survival status are presented in Table 1.

### 3.2. Comparison Between Survivors and Non-Survivors

Non-survivors had a significantly higher prevalence of cardiovascular disease and dyspnea at admission, as well as a higher likelihood of hospital-origin admission compared to survivors (Table 1).

### 3.3. Factors Associated with Mortality

In exploratory multivariable logistic regression analysis, cardiovascular disease, chronic respiratory disease, dyspnea, and hospital-origin admission were associated with in-hospital mortality (Table 2).

To assess potential multicollinearity among predictors included in the multivariable logistic regression model, VIFs were calculated. All VIF values were below 1.5, indicating minimal correlation among explanatory variables. The highest VIFs were observed for age (1.32) and cardiovascular disease (1.35), well below commonly used thresholds for concern (>5). Multimorbidity showed a VIF of 1.0, indicating no correlation with other predictors. These results suggest that multicollinearity did not significantly influence the estimated odds ratios (Table 3).

### 3.4. Dialysis Vintage and Mortality

**Figure 2:**
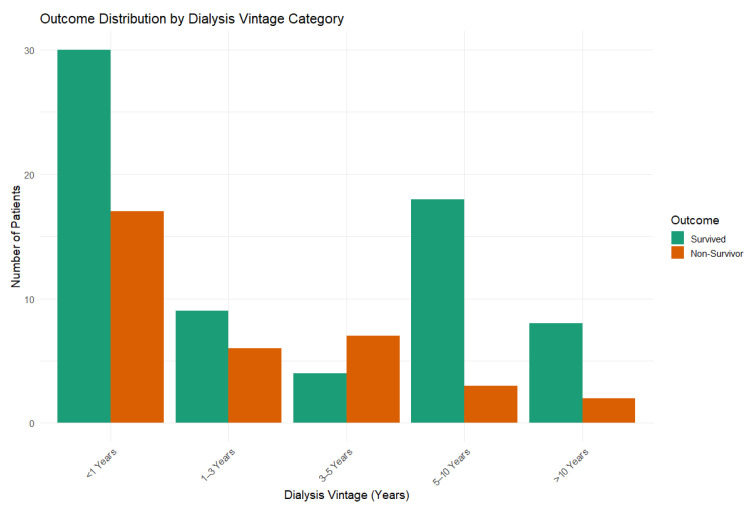
In-hospital mortality according to dialysis vintage categories.

Dialysis vintage data were available for a subset of patients (n = 113). The distribution of in-hospital mortality across dialysis vintage categories is shown in Figure 2. No clear linear association between dialysis vintage duration and in-hospital mortality was observed, with deaths occurring across all vintage categories. Due to the exploratory nature of this analysis and incomplete data, dialysis vintage was not included in the multivariable regression model.

### 3.5. Length of Hospital Stay

**Figure 3:**
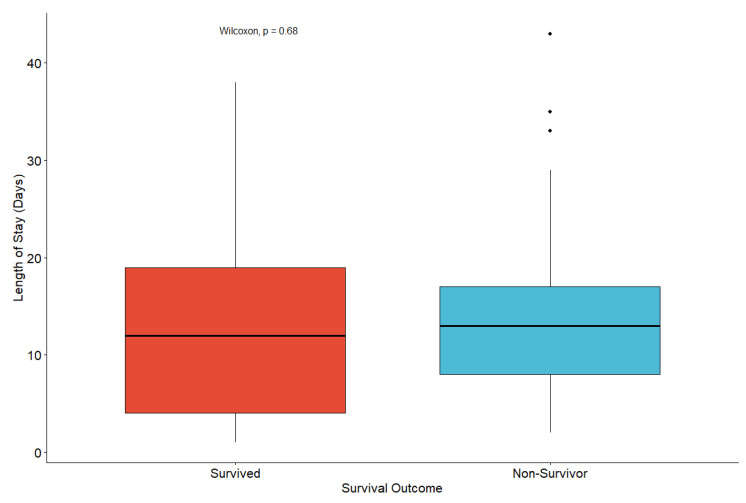
Length of hospital stay according to survival outcome (Wilcoxon test).

No significant difference in length of hospital stay was observed between survivors and non-survivors (median 12 vs. 13 days, Wilcoxon *p* = 0.68), as illustrated in Figure 3.

## 4. Discussion

The analysis of 130 hemodialyzed patients diagnosed with COVID-19 included in our study highlights distinct clinical and prognostic features compared to the general population infected with SARS-CoV-2. The mean age of 64 years (±13.9) and the predominance of males (64.6%) confirm the international trend that advanced age and male sex are major risk factors for severe evolution and increased mortality among dialysis patients [26,27]. Similarly to other studies, elderly patients from our cohort, particularly those over 70 years old, had a significantly higher rate of respiratory complications, requiring non-invasive ventilation or prolonged oxygen therapy. This was further reflected in prolonged hospitalizations and fatal outcomes, confirming that age-associated frailty profoundly limits recovery potential in this population [14,28].

Beyond descriptive characterization, comparative analyses between survivors and non-survivors and logistic regression modeling were performed to identify independent predictors of mortality in this cohort. The observed mortality rate in our cohort (34.6%) is consistent with internationally reported data. The ERACODA study, which evaluated a cohort of 2449 hemodialysis patients, reported survival probabilities of 90%, 73%, and 40% for non-hospitalized, hospitalized, and intensive care unit (ICU) patients, respectively, three months after COVID-19 diagnosis [14]. These findings suggest that early mortality is concentrated among the most vulnerable patients, while functional recovery among survivors may remain relatively favorable. Consistent with ERACODA data, our results further indicate that age, frailty, and severe clinical presentation contribute to diminished physiological resilience under multiorgan stress.

Comparative analysis between survivors and non-survivors revealed that several clinical characteristics differed significantly between outcome groups. Cardiovascular disease and dyspnea were significantly more frequent among non-survivors, while hospital-origin admission was also associated with worse outcomes. In contrast, age, sex, diabetes mellitus, and length of hospital stay did not show statistically significant differences between survivors and non-survivors, suggesting that mortality was driven more by baseline vulnerability and acute disease severity rather than hospitalization duration alone.

Multivariable logistic regression analysis further refined these observations by identifying independent predictors of mortality. Cardiovascular disease, chronic respiratory disease, dyspnea at presentation, and hospital-origin admission emerged as significant predictors of fatal outcome. Dyspnea demonstrated the strongest association with mortality, reflecting advanced respiratory involvement and severe disease at presentation. Although hypertension and diabetes mellitus were highly prevalent in the cohort, they were not independently associated with mortality after adjustment for confounding factors, underscoring the importance of cumulative cardiovascular and respiratory burden rather than isolated metabolic conditions. Interestingly, arterial hypertension showed an apparent protective association in multivariable analysis. This finding should be interpreted with caution and may reflect the predominance of acute clinical severity markers, such as dyspnea at presentation and hospital-origin admission, which likely overshadow the prognostic contribution of chronic conditions once included in the model. In addition, this observation is consistent with the concept of “reverse epidemiology” in patients undergoing maintenance hemodialysis, in which traditional cardiovascular risk factors, such as hypertension, paradoxically associate with improved survival [29,30].

Although dialysis vintage is a well-recognized prognostic factor in end-stage renal disease, particularly in long-term outcome analyses, our exploratory analysis did not demonstrate a clear association between dialysis duration and in-hospital mortality. This finding contrasts with larger cohorts evaluating long-term survival, where dialysis vintage has been shown to influence 5-year mortality and cardiovascular outcomes [31]. However, these differences are likely explained by the distinct clinical context addressed in the present study. In acute COVID-19–related hospitalization, short-term mortality appears to be predominantly driven by markers of acute disease severity and cumulative cardiopulmonary burden rather than by long-term dialysis exposure.

The high prevalence of cardiovascular comorbidities and diabetes mellitus among the analyzed patients aligns with data from European multicenter studies [15,25]. These conditions are recognized as negative prognostic factors through their direct impact on vascular and metabolic systems and through impaired immune responses to viral infection. Our findings are consistent with reports from the ERA-EDTA COVID-19 Database, which documented mortality rates exceeding 25% among diabetic dialysis patients during the pandemic peak [24]. Moreover, the association between cardiovascular disease and poor prognosis has been widely reported, with older age, heart disease, and frailty consistently identified as critical predictors of adverse outcomes [18,21].

Multimorbidity burden, defined as the presence of 2 or more chronic conditions, was common in our cohort and was associated with increased mortality risk in unadjusted analyses. In multivariable analysis, the association between multimorbidity (≥2 chronic conditions) and mortality did not reach statistical significance, with a very wide confidence interval indicating limited precision, likely due to the small number of patients without multimorbidity. Nevertheless, this observation supports the hypothesis that cumulative cardiovascular, metabolic, and respiratory disorders heighten vulnerability to cytokine storm, endothelial dysfunction, and microvascular injury in patients undergoing maintenance hemodialysis.

The mean hospitalization duration of 13.6 ± 9.7 days reflects the clinical severity and management complexity of this population. Consistent with large multicenter and meta-analytic data, SARS-CoV-2–positive dialysis patients experience significantly longer hospital stays than patients without end-stage kidney disease, with typical lengths of stay ranging from 11 to 15 days [18,22,32,33]. In our cohort, length of stay did not differ significantly between survivors and non-survivors, suggesting that fatal outcomes were primarily influenced by baseline comorbidities and acute disease severity rather than prolonged hospitalization per se.

The predominant symptomatology observed in our cohort, fever, dyspnea, and fatigue, mirrors international data. However, the high incidence of severe respiratory involvement among non-survivors is consistent with the inflammatory and metabolic dysregulation associated with chronic uremia. Previous studies have reported that 6.1–35.7% of dialysis patients with COVID-19 developed respiratory distress requiring ICU admission and mechanical ventilation [17]. Immune dysregulation in dialysis populations [34], together with longer dialysis vintage and cumulative treatment exposure, may further contribute to unfavorable outcomes [25,35,36,37].

In the context of the pandemic, the literature has emphasized the vulnerability of dialysis patients to nosocomial transmission, particularly in dialysis centers [38]. In our study, a substantial proportion of patients were admitted from hospital settings, reinforcing the importance of strict infection control measures. Early implementation of isolation, triage, and personal protective equipment protocols likely contributed to reduced incidence of severe cases during later stages of the study period. Similar nationwide trends were reported in Romania, where pandemic-related healthcare disruption increased nosocomial transmission risks [39].

This study has several limitations that should be acknowledged. First, the relatively small sample size may limit the ability to detect smaller, yet clinically relevant, associations. In this context, although the number of variables included in the multivariable model was relatively high in relation to the sample size, formal assessment of multicollinearity using variance inflation factors showed minimal correlation among predictors. All VIF values were below commonly accepted thresholds, suggesting that multicollinearity was unlikely to substantially bias the estimated associations; however, residual confounding and limited statistical power cannot be fully excluded. Second, the retrospective design is inherently subject to selection bias and relies on the accuracy and completeness of medical records. Third, data were obtained from a single large tertiary nephrology center, which may limit the generalizability of the findings to other healthcare systems or regions. Additionally, the cohort included only patients admitted to a nephrology unit, potentially excluding milder cases managed in outpatient settings and terminal cases that occurred prior to hospital admission, which may have influenced the observed mortality rate. The study period (March 2020–April 2021) encompassed different phases of the pandemic, including evolving viral variants and treatment protocols, which were not analyzed separately. Furthermore, although major comorbidities were included in the multivariable models, vaccination status were not consistently available in the retrospective medical records and could therefore not be analyzed. Finally, hospital versus ambulatory origin reflects the clinical context at the time of COVID-19 diagnosis, with hospital-origin cases representing patients diagnosed during hospitalization for other conditions or transferred due to disease severity, which may partly explain the higher mortality observed in this group.

Importantly, the findings of this study should be interpreted within the specific temporal context of the early COVID-19 pandemic. The study period preceded the widespread availability of vaccination and the emergence of less virulent SARS-CoV-2 variants, both of which have substantially modified the clinical course and outcomes of COVID-19 in the general population. Consequently, the present results should not be viewed as representative of the current epidemiological landscape, but rather as providing historical and contextual insight into mortality determinants during a phase of maximal disease severity, particularly in highly vulnerable populations such as patients undergoing maintenance hemodialysis.

Altogether, these findings emphasize the importance of early risk stratification in hemodialysis patients with COVID-19, with particular attention to cardiovascular comorbidities, respiratory involvement at presentation, and hospital-origin admission. Identifying high-risk patients early may facilitate timely escalation of care, closer monitoring, and implementation of tailored therapeutic strategies aimed at reducing mortality and optimizing resource allocation in this highly vulnerable population.

## 5. Conclusions

This study confirms that patients undergoing chronic hemodialysis represent a particularly vulnerable population in the context of COVID-19 infection, with a high burden of complications and mortality compared to the general population. Advanced age and a high prevalence of cardiovascular and respiratory comorbidities characterize this group and are consistent with international reports describing mortality rates of approximately 25–30% among dialysis patients infected with SARS-CoV-2.

Importantly, our analyses identified cardiovascular disease, chronic respiratory disease, dyspnea at presentation, and hospital-origin admission as key factors associated with mortality, highlighting the combined impact of baseline cardiorespiratory vulnerability and disease severity. While metabolic conditions such as diabetes mellitus and hypertension were highly prevalent, they did not independently predict mortality after adjustment, suggesting that cumulative comorbidity burden and organ-specific dysfunction play a more decisive prognostic role. Although dialysis vintage is a recognized prognostic factor in end-stage renal disease, exploratory analyses in our cohort did not identify a clear association with in-hospital mortality, suggesting that short-term outcomes in the context of acute COVID-19 infection are predominantly driven by acute clinical severity and cumulative cardiopulmonary burden rather than long-term dialysis exposure.

The observed outcomes likely reflect impaired immune response, persistent systemic inflammation, and unavoidable exposure during dialysis procedures. These findings emphasize the need for strict infection control measures in dialysis centers, early risk stratification, and close clinical monitoring. Targeted surveillance and timely intervention in high-risk patients may help reduce mortality in this particularly vulnerable population.

## Figures and Tables

**Table 1:** Baseline clinical characteristics stratified by survival status.

Characteristic	Survived N = 83 ^1^	Non-Survivor N = 45 ^1^	*p*-Value ^2^
Age (years)	65 (54, 71)	69 (55, 78)	0.13
Gender			0.4
Male	56 (67%)	26 (58%)	
Female	27 (33%)	19 (42%)	
Hypertension	66 (80%)	28 (62%)	0.057
Diabetes	37 (45%)	24 (53%)	0.4
CVD	37 (45%)	31 (69%)	0.014
Respiratory	11 (13%)	13 (29%)	0.054
Multimorbidity (≥2)			0.3
<2	4 (4.8%)	0 (0%)	
≥2	79 (95%)	45 (100%)	
Fever	17 (20%)	15 (33%)	0.2
Dyspnea	19 (23%)	26 (58%)	<0.001
Fatigue	14 (17%)	5 (11%)	0.5
Origin			0.005
Hospital	46 (55%)	37 (82%)	
Ambulatory	37 (45%)	8 (18%)	
Length of Stay (days)	12 (4, 19)	13 (8, 17)	0.7

^1^ Median (Q1, Q3); n (%); ^2^ Wilcoxon rank sum test; Pearson’s Chi-squared test.

**Table 2:** Factors associated with in-hospital mortality: logistic regression analysis.

Characteristic	N	OR	95% CI	*p*-Value
Age (years)	128	1.01	0.99, 1.04	0.3
Gender	128			
Male		—	—	
Female		1.49	0.71, 3.21	0.3
Hypertension	128			
No		—	—	
Yes		0.44	0.19, 0.95	0.041
Diabetes	128			
No		—	—	
Yes		1.40	0.69, 2.96	0.4
CVD	128			
No		—	—	
Yes		2.64	1.30, 6.06	0.011
Respiratory	128			
No		—	—	
Yes		2.52	1.08, 6.69	0.039
Multimorbidity (≥2)	128			
<2		—	—	
≥2		6.61	0.35, 125	0.2
Fever	128			
No		—	—	
Yes		1.88	0.85, 4.42	0.12
Dyspnea	128			
No		—	—	
Yes		4.36	2.13, 10.3	<0.001
Fatigue	128			
No		—	—	
Yes		0.64	0.19, 1.75	0.4
Origin	128			
Hospital		—	—	
Ambulatory		0.29	0.11, 0.62	0.004
Length of Stay (days)	128	1.00	0.96, 1.04	>0.9

CI = Confidence Interval, OR = Odds Ratio.

**Table 3:** Variance inflation factors (VIFs) for predictors included in the multivariable logistic regression model.

Variance Inflation Factors Assessment of Multicollinearity	
Variable	VIF
Age	1.324734
Sex	1.094330
LOS	1.144425
Origin	1.159398
is_HTA	1.103522
is_Diabetes	1.071242
is_CVD	1.350901
is_Resp	1.133757
Multimorbidity	1.000000
Symp_Fever	1.274696
Symp_Dyspnea	1.158186
Symp_Fatigue	1.181232

## Data Availability

The original contributions presented in this study are included in the article. Further inquiries can be directed to the corresponding author.

## Abbreviations

The following abbreviations are used in this manuscript:

COVID-19	coronavirus disease 19
SARS-CoV-2	severe acute respiratory syndrome coronavirus 2
ACE2	angiotensin-converting enzyme II
ESRD	end-stage renal disease
TMPRSS2	transmembrane serine protease 2
CKD	chronic kidney disease
ERACODA	European Renal Association COVID-19 Database
ORs	Odds ratios
CIs	confidence intervals
